# The Specific Impacts of Allelopathy and Resource Competition from *Artemisia frigida* on the Growth of Three Plant Species in Northern China

**DOI:** 10.3390/plants13233286

**Published:** 2024-11-22

**Authors:** Qing Wang, Mengqiao Kong, Junwen Wang, Bin Gao, Xiaoyan Ping

**Affiliations:** 1 School of Grassland Science, Beijing Forestry University, No. 35 Qinghua East Road, Haidian District, Beijing 100083, China; wangqingi2020@163.com (Q.W.); zhforeverj@163.com (M.K.); jwwang2022@163.com (J.W.); ibcas2011@163.com (B.G.); 2 College of Grassland Science and Technology, China Agricultural University, No. 2 Yuanmingyuan West Road, Haidian District, Beijing 100193, China

**Keywords:** *Artemisia frigida*, Eurasian Steppe, grazing intensity, outdoor pot experiment, plant interference

## Abstract

Plant interference is a key factor influencing plant coexistence and species composition. The two primary forms of plant interference—allelopathy and resource competition—are often difficult to separate. This study conducted an outdoor pot experiment to quantify the distinct contributions of resource competition and allelopathy of *Artemisia frigida* on seedling growth of three species: *Leymus chinensis*, *Cleistogenes squarrosa*, and *Potentilla acaulis*. The index of relative neighbor effect (*RNE*) was used to quantify the overall effect of plant interference, while the inhibition rates (*IRs*) of resource competition and allelopathy were utilized to determine the specific contributions of allelopathy and resource competition from *A*. *frigida* on the growth of target plant species. The interference effect of *A*. *frigida* was found to be species-specific. The allelopathic effect of *A*. *frigida* played a major role in inhibiting the belowground biomass of *L*. *chinensis* (23.97%) and *C*. *squarrosa* (58.27%), while allelopathy and resource competition from *A*. *frigida* promoted the belowground biomass (45.12%) and aboveground biomass (46.63%) of *P*. *acaulis*, respectively. The combined effect of allelopathy and resource competition from *A*. *frigida* significantly affected the aboveground biomass of *C*. *squarrosa* and *P*. *acaulis*, as well as the belowground biomass of *L*. *chinensis* and *C*. *squarrosa*. These findings contribute to a better understanding of the patterns and mechanisms of plant species composition and its relationship with grazing intensity in this grassland ecosystem.

## 1. Introduction

Plant interference is a central driver for plant coexistence [1]. The negative interspecific interaction can be attributed to resource competition (competing for water, nutrients, light, or space) or allelopathy (chemical interactions among species) [2,3,4]. Allelopathy can alter the dynamics of resource competition by influencing the growth of recipient species [5,6], while the intensity of resource competition can also affect the release of allelochemicals [7,8]. Traditionally, resource competition has been considered the primary driver of plant community diversity and dynamics [9]. However, other researchers have found that allelopathy can significantly influence plant community composition and succession, potentially playing a role as crucial as resource competition [4,10,11].

Numerous studies have been conducted to distinguish between the two types of plant interference [7,8,12]. For example, PVC tubes have been employed to mitigate both aboveground and root competition between two species, while activated carbon (AC) has been utilized to absorb and eliminate allelochemicals leached by donor plants [12,13,14]. Partial least squares regression or glasshouse experiments have been employed to ascertain the relative contribution of resource competition and allelopathy on the growth of winter cereals and rice [15,16]. Density-dependent experiments have been conducted to quantify the relative contributions of allelopathy and competition to overall interference [2,3,17], while some research has focused on the impact of competition and allopathy on community composition in coastal and inland ecosystems [18]. However, nature is inherently complex, where allelopathy and resource competition often coexist and interact closely [19,20]. The specific contribution of these two types of plant interference remains unknown.

Grasslands constitute approximately 40% of the global land area, offering a plethora of benefits to over 1 billion people worldwide [21]. The most prevalent and significant use of grasslands is for livestock grazing [22,23]. Extensive research has shown that plant species composition varies considerably along grazing gradients [24,25]. *Artemisia frigida* Willd. (Prairie Sagebrush) is a resilient perennial semi-shrub found throughout the Eurasian Steppe. It exhibits remarkable resistance to drought, grazing, trampling, and regeneration and can be used as high-quality forage, especially in spring and winter [22,23,25]. Meanwhile, *Artemisia frigida* is the dominant species under moderate and heavy grazing intensities in temperate grasslands of northern China [22]. Conversely, *Leymus chinensis* prevails in the eastern Eurasian Steppe, while *Cleistogenes squarrosa* and *Potentilla acaulis* are the dominant species under light and extreme grazing intensities, respectively [25]. *Cleistogenes squarrosa* is a C_4_ perennial bunchgrass noted for its robust drought tolerance, whereas *P*. *acaulis* is a perennial stoloniferous herb known for its pronounced allelopathy [22,24]. Both *A. frigida* and *P*. *acaulis* exhibit allelopathic effect, and the aqueous extracts from these two species and the volatile organic compounds (VOCs) from *A*. *frigida* markedly inhibit the growth of *C*. *squarrosa* and *L*. *chinensis* [22,25,26]. The ecological adaptation of *A*. *frigida* to prolonged continuous grazing can be attributed to its strong phenotypic plasticity [27], altered carbon allocation patterns [28], intense interspecific competitiveness [24], and allelopathy [22,25,29,30].

To assess the distinct impact of resource competition and allelopathy of *A*. *frigida* on the growth of common species within the Eurasian Steppe, we initiated an outdoor pot experiment in northern China. We hypothesized that the plant interference of *A*. *frigida* would exhibit species-specific variation among different plant species. Our study sought to address the following question: How does the interference from *A*. *frigida* differ across various target plant species, and how is this interference influenced by grazing intensity and specific plant species?

## 2. Results

### 2.1. Interference Between A. frigida and Three Target Plant Species

For the interference of *A*. *frigida* with three target plant species (AF-LC, AF-CS, and AF-PA in Table 1), *A*. *frigida* inhibited both the aboveground (LC 0.28, CS 0.45) and belowground biomass (LC 0.15, CS 0.64) of *L*. *chinensis* and *C*. *squarrosa* but promoted the root/shoot ratio (−0.16) of *L*. *chinensis*. Concurrently, *A*. *frigida* enhanced the aboveground biomass (−0.08), belowground biomass (−0.67), and root/shoot ratio (−0.64) of *P*. *acaulis*. The index of relative neighbor effect (*RNE)* of the aboveground biomass, belowground biomass, and root/shoot ratio were all significantly different among the three target species (aboveground biomass: *df* = 8, *F* = 19.875, *P* = 0.002; belowground biomass: *df* = 8, F = 535.398, *P* < 0.001; root/shoot ration: *df* = 8, F = 59.108, *P* < 0.001). Notably, the growth inhibitory effect of *A*. *frigida* on *C*. *squarrosa* was stronger, especially in terms of belowground biomass and root/shoot ratio, when compared to *L*. *chinensis*.

As for the interference of three target plant species with *A*. *frigida* (LC-AF, CS-AF and PA-AF in Table 1), in addition to the inhibitory effect of *P*. *acaulis* on the aboveground biomass (0.30) of *A*. *frigida*, the other two target plant species all promoted the aboveground biomass (LC −0.27, CS −0.64) and belowground biomass (LC −0.40, CS −0.52) of *A*. *frigida*. Meanwhile, the promoting effects of *C*. *squarrosa* on the aboveground and belowground biomass of *A*. *frigida* were both significantly greater than that of *L*. *chinensis* (*df* = 5, *F* = 136.862, *P* = 0.001 and *df* = 5, *F* = 18.752, *P* = 0.012).

### 2.2. Specific Contributions of Allelopathy and Resource Competition from A. frigida to the Growth of Three Target Species

**Figure 1:**
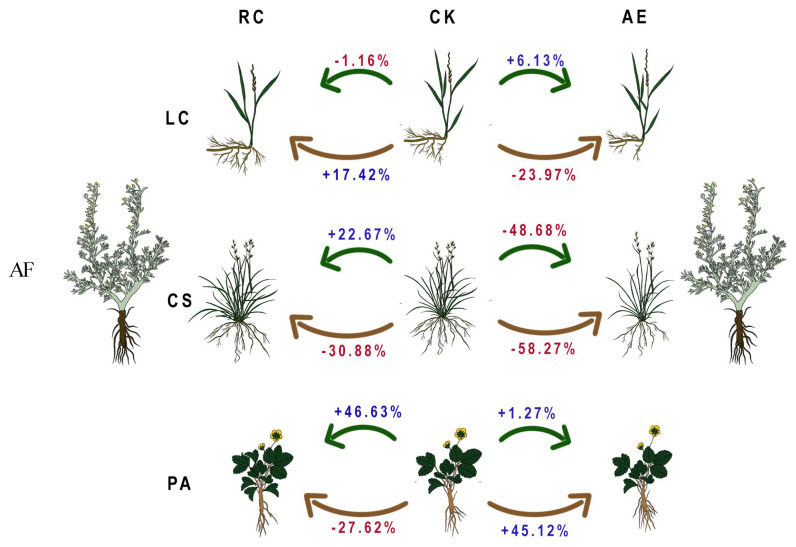
Specific contribution of allelopathy and resource competition from *Artemisia frigida* to the growth of three target species (LC: *Leymus chinensis*, AF: *Artemisia frigida*, CS: *Cleistogenes squarrosa*, PA: *Potentilla acaulis*; RC: resource competition; AE: allelopathy; and CK: monoculture. Red font in the figure indicates a growth-inhibiting effect, while blue font indicates a growth-promoting effect. The green arrows in the figure indicate aboveground biomass, and the brown arrows indicate belowground biomass).

For *L*. *chinensis*, allelopathy of *A*. *frigida* played a predominant role in plant interference, particularly affecting the belowground biomass (Figure 1). The resource competition from *A*. *frigida* led to a decrease in the aboveground biomass by 1.16% but increased the belowground biomass by 17.42%. Furthermore, the allelopathy of *A*. *frigida* resulted in a reduction of the belowground biomass by 23.97% yet augmented the aboveground biomass by 6.13%.

For *C*. *squarrosa*, the allelopathy exhibited by *A*. *frigida* played a major role in affecting its aboveground and belowground biomass. Specifically, resource competition initiated by *A*. *frigida* resulted in a 22.67% increase in the aboveground biomass while simultaneously causing a 30.88% decrease in the belowground biomass. Conversely, the allelopathy of *A*. *frigida* resulted in a reduction in the aboveground biomass by 48.68% and the belowground biomass by 58.27%.

As for *P*. *acaulis*, both the aboveground and belowground biomass was affected by the resource competition from *A*. *frigida*. This competition led to a 46.63% increase in the aboveground biomass and a 27.62% decrease in the belowground biomass. Additionally, the allelopathy of *A*. *frigida* contributed to a 1.27% increase in the aboveground biomass and a 45.12% increase in the belowground biomass of *P*. *acaulis*.

### 2.3. Effects of Allelopathy and Resource Competition and Their Coupling Effect on the Plant Growth of Three Target Species

**Figure 2:**
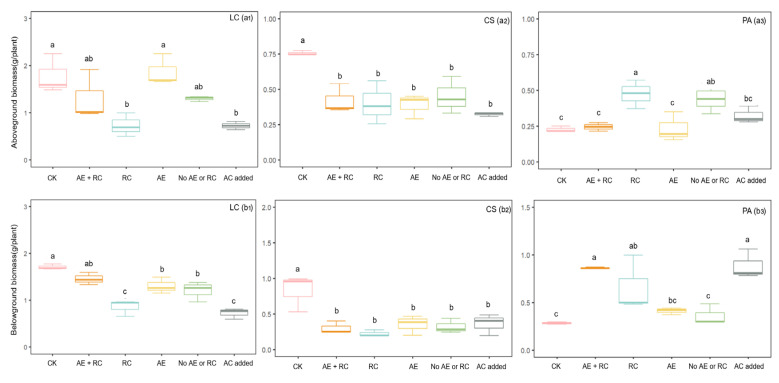
Effects of allelopathy and resource competition of *Artemisia frigida* on the growth of three target plant species (All the data are shown as mean ± SE (n = 3). (**a**): Effects of allelopathy and resource competition of *Artemisia frigida* on the aboveground biomass of *L*. *chinensis* (**a1**), *C*. *squarrosa* (**a2**), *P*. *acaulis* (**a3**). (**b**): Effects of allelopathy and resource competition of *Artemisia frigida* on the belowground biomass of *L*. *chinensis* (**b1**), *C*. *squarrosa* (**b2**), *P*. *acaulis* (**b3**). CK: monoculture; AE + RC: both allelopathy and resource competition; RC: resource competition; AE: allelopathy of *A*. *frigida*; No AE or RC: neither allelopathy nor resource competition of *A*. *frigida*; and AC added: activated carbon was added to evaluate the specific impact of activated carbon on plant growth of three target plant species). Different lowercase letters show the significant differences in biomass of the same plant specie between different treatments (*P* < 0.05).

The allelopathic effect (AE vs. CK) of *A*. *frigida* significantly reduced the belowground biomass of *L*. *chinensis* (*df* = 17, *F* = 17.319, *P* < 0.001), while resource competition (RC vs. AC added) did not have a significant impact (Figure 2(a1,b1)). Neither factor significantly affected the aboveground biomass of *L*. *chinensis* (Figure 2(a1)). The combined effect of allelopathy and resource competition of *A*. *frigida* did not significantly influence either the aboveground or belowground biomass of *L*. *chinensis* (AE + RC vs. CK). However, the addition of activated carbon significantly inhibited the aboveground (*df* = 5, *F* = 18.572, *P* = 0.013) and belowground biomass (*df* = 5, *F* = 170.763, *P* < 0.001) of *L*. *chinensis* (AC added vs. CK).

The allelopathic effects of *A*. *frigida* significantly reduced both the aboveground (*df* = 17, *F* = 7.083, *P* = 0.003) and belowground biomass (*df* = 17, *F* = 6.529, *P* = 0.004) of *C*. *squarrosa* (Figure 2(a2,b2)). Conversely, resource competition from *A*. *frigida* did not have a significant effect on either the aboveground or belowground biomass of *C*. *squarrosa*. However, when considering the combined effects of allelopathy and resource competition from *A*. *frigida*, there was a significant inhibition observed in both the aboveground (*df* = 5, *F* = 30.536, *P* = 0.005) and belowground biomass (*df* = 5, *F* = 11.170, *P* = 0.029) of *C*. *squarrosa*. Furthermore, the introduction of activated carbon led to a significant reduction in the aboveground (*df* = 5, *F* = 134.602, *P* < 0.001) and belowground biomass (*df* = 5, *F* = 8.114, *P* = 0.046) of *C*. *squarrosa*.

The allelopathic effects of *A*. *frigida* did not significantly affect the aboveground and belowground biomass of *P*. *acaulis* (Figure 2(a3,b3)). However, resource competition from *A*. *frigida* significantly enhanced the aboveground biomass of *P*. *acaulis* (*df* = 17, *F* = 5.870, *P* = 0.006). The combined effect of allelopathy and resource competition from *A*. *frigida* significantly increased the belowground biomass of *P*. *acaulis* (*df* = 17, *F* = 10.162, *P* = 0.001) (Figure 2(b3)). The addition of activated carbon significantly increased the belowground biomass of *P*. *acaulis* (*df* = 5, *F* = 45.518, *P* = 0.003).

**Figure 3:**
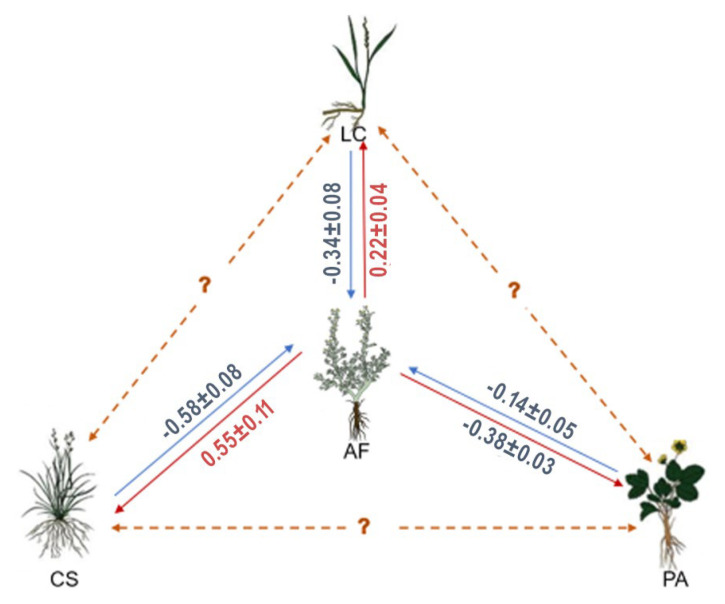
Interference of *Artemisia frigida* with the plant growth of three target species (We used the average *RNE* of aboveground and belowground biomass to represent the interference of *A*. *frigida* with three target species (red arrows). Meanwhile, the interference of three target species with *A*. *frigida* was also marked in the figure (blue arrows). The blue values signify a promotional effect, while the red values denote an inhibitory effect).

Our study found that, compared to monocultured *L*. *chinensis* and *C*. *squarrosa*, the interference of *A*. *frigida* inhibited the plant growth of these species (LC 0.22, CS 0.55). Conversely, the interference of *A*. *frigida* promoted the growth of *P*. *acaulis* compared to its monoculture condition (−0.38). However, the interference from *L*. *chinensis*, *C*. *squarrosa*, and *P*. *acaulis* all had a positive effect on the growth of *A*. *frigida* (LC −0.34, CS −0.58, PA −0.14) (Figure 3).

## 3. Discussion

### 3.1. Interference of A. frigida with Three Target Plant Species

Our results support our hypothesis that the interference of *A*. *frigida* was species-specific. However, this study only examined the pairwise interference of *A*. *frigida* on three target plant species, leaving the specific interference of *L*. *chinensis*, *C*. *squarrosa* and *P*. *acaulis* to each other unexplained (Figure 3). The impact of interference on phenotypic plasticity can have more profound effects on species composition than above- and belowground biomass. For instance, the inhibitory effect of *Plantago lanceolate* on the root growth of *Festuca rubra* could diminish the competitive strength of *F*. *rubra* against other species within the community [31]. Similarly, the inhibitory effect of *P*. *acaulis* on the plant growth of *A*. *frigida* may alter the interference of *A*. *frigida* with other plant species at the study site (Figure 3). Therefore, to gain a comprehensive understanding of the role of plant interference on species composition, it is necessary to consider not only pairwise interactions but also the interaction chain among coexisting plant species in future research.

The interaction between two species may be conditional, depending on environmental conditions, and herbivory may also change the balance of interference among species [32]. In a multispecies community, the species interactions are the sum of all intraspecific and interspecific interactions. Moreover, the interference among species is important in shaping community dynamics and maintaining biodiversity [33]. Concurrently, plant interference may also be influenced by plant–soil feedbacks (PSFs), which can promote the growth of neighboring species in heterogeneous environments [34]. Furthermore, competition among species within the same clades tends to favor dissociation, primarily due to asymmetrical competition within the community [35]. Species with high interspecific competition ability along with grazing intensity would result in asymmetric competition. Highly dominated communities are often characterized by strongly asymmetric competition [36]. Our findings indicate that *A*. *frigida* and *P*. *acaulis* possess a relatively high degree of interspecific competition ability in comparison to *L*. *chinensis* and *C*. *squarrosa* (Figure 3). This asymmetrical competition could potentially lead to the dominance of *A*. *frigida* and *P*. *acaulis* under conditions of heavy and extreme grazing.

### 3.2. Separation of Allelopathy and Resource Competition from Plant Interference

Consistent with prior research, our findings validated that the plant interference of *A*. *frigida* was a synergistic effect of resource competition and allelopathy [17]. Furthermore, we established that the combined effect of allelopathy and resource competition significantly impacted plant–plant interference, with the primary type and intensity of interference from *A*. *frigida* varying among different plant species. The allelopathic effect of *A*. *frigida* became important for *L*. *chinensis* and *C*. *squarrosa* under conditions of no grazing and light grazing intensity (Figure 2). Meanwhile, both the allelopathy and resource competition of *A*. *frigida* facilitated the dominance of *P*. *acaulis* under extreme grazing intensity. Therefore, this study provides a partial explanation for the mechanism of community succession in temperate grasslands along grazing gradients.

Several methods have been developed to distinguish resource competition from allelopathy [8,19,37]. However, most prior research has overlooked the combined effect of allelopathy and resource competition [38]. This study addresses two significant limitations in allelopathy research. Firstly, we implemented a field control experiment, enhancing the credibility of our results compared to those derived from greenhouse experiments. Secondly, this study not only isolates the allelopathic effects of *A*. *frigida* from resource competition but also examines the interactive effect of resource competition and allelopathy of *A*. *frigida* on the plant growth of three target plant species.

The application of activated carbon (AC) has been widely utilized in allelopathy studies to adsorb allelochemicals and mitigate soil toxicity caused by these compounds. This method effectively separates the effects of allelopathy from resource competition [39,40]. However, the use of AC can introduce controversy due to potential side effects such as altering soil nutrient availability and plant growth [40] and affecting soil microbial activity [41]. Our study confirmed that the addition of AC can influence the plant growth of target plant species, although the specific effects varied among the three target plant species (Figure 2). In contrast to the inhibitory effect of AC on the plant growth of *L*. *chinensis* and *C*. *squarrosa* (Figure 2), the addition of AC significantly promoted the plant growth of *P*. *acaulis* (Figure 2). This discrepancy could be attributed to the fact that AC may also absorb the allelochemicals released by *P*. *acaulis* itself, which has been shown to have strong allopathic effects on the seed germination of *L*. *chinensis* and *A*. *frigida* [26]. Additionally, we used AC added to *L*. *chinensis*, *C*. *squarrosa*, and *P*. *acaulis* as controls to assess the impact of resource competition on the plant growth of three target plant species. By incorporating controls for the effect of AC in our experimental design, our results provide a clearer reflection of the relative contributions of allelopathic interference and resource competition to plant growth.

### 3.3. The Impact of Allelopathy and Resource Competition and Their Coupling Effects on the Dominant Status of A. frigida in Moderate Grazing Grassland

Our findings corroborate previous research indicating that allelopathy can vary among target plant species, either stimulating or inhibiting growth [42]. For instance, the aboveground biomass of *L*. *chinensis* was marginally enhanced by the allelopathy of *A*. *frigida*, while the belowground biomass was significantly suppressed [43,44]. In this study, *C*. *squarrosa* demonstrated a higher sensitivity to allelopathy of *A*. *frigida* than *L*. *chinensis*, contradicting previous results [22]. This discrepancy may be attributed to *C*. *squarrosa*’s adaptability to environments with lower precipitation levels; thus, its interference in the community is likely to increase under conditions of aridity induced by livestock grazing [45]. The heavy rainfall during the latter stages of our experiment may have heightened *C*. *squarrosa*’s sensitivity to both the allelopathy and resource competition of *A*. *frigida*. Additionally, the belowground biomass was more significantly affected by the allelopathy of *A*. *frigida*, aligning with previous findings in northern China [22].

Allelopathy, whereby the growth of neighboring plants is inhibited by donor plants, can simultaneously enhance their resource competitiveness [4]. This form of intransitive competition can lead to changes in plant species composition [33]. Our study found that the combined effect of allelopathy and resource competition significantly influenced the aboveground biomass of *P*. *acaulis* and the above- and belowground biomass of *C*. *squarrosa* (Figure 2). However, we were unable to precisely quantify the contribution of this coupling effect. Our results indicated that the coupling effect varied substantially among the three target plant species. It could increase the above- and belowground biomass of *L*. *chinensis* and *C*. *squarrosa* and the belowground biomass of *P*. *acaulis*, and it could also decrease the aboveground biomass of *P*. *acaulis*. Therefore, the coupling effect of allelopathy and resource competition could either offset or amplify the actual effect, playing an important role in regulating species composition at the study site [14].

Allelopathy can facilitate the dominance of specific plants within a community, especially for alien plants [6]. Additionally, allelopathy has been identified as a key factor driving grassland succession in northern China [22,44] and forest dynamics in Mediterranean and southern China [11,46]. Resource competition is widely recognized as one of the most significant mechanisms of plant interference in shaping species composition. Concurrently, previous research has indicated that the competition among plant species in the absence of herbivores transitions to facilitation in heavily grazed pastures [47]. In stressful habitats, positive neighbor effects become increasingly important for plant interference [48], a phenomenon that may also be reflected in our study. Along with grazing gradients, the high intensity of trampling and feeding imposed by livestock can cause mechanical damage to plants of *A*. *frigida* and increase the release of allelochemicals [49]. A prior study revealed that grazing intensity could influence the components and concentrations of allelochemicals released by *A*. *frigida*, with the maximum relative contents of 1,8-cineole and β-terpineol both occurring under heavy grazing intensity [25]. These high concentrations of allelochemicals had a strong inhibitory effect on the plant growth of *C*. *squarrosa* and a promotional effect on the plant growth of *P*. *acaulis*. However, we did not take into account the concentration of allelochemicals, particularly the VOCs released by *A*. *frigida*, throughout the entire experiment, which should be considered in future research.

## 4. Materials and Methods

### 4.1. Study Site

This study was conducted at the Hulunbuir field observation experimental station (48°55′48″ N, 119°41′26″ E, altitude 636 m) of the Chinese Academy of Environmental Sciences in Temo Huzhu, situated in the Ewenki Autonomous Banner of Hulunbuir city, China. The study site is characterized by a mid-temperate continental monsoon climate, with an annual average temperature of −2.4 to 2.2 °C and an annual average precipitation from 330 to 350 mm (mainly occurring from June to August). The grassland at the study site is part of the Eurasian Steppe, and the soil is classified as calcic chestnuts, equivalent to Kastanozems according to the soil classification system of the Food and Agriculture Organization (FAO). In the absence of grazing, *L*. *chinensis* and *Stipa baicalensis* are the dominant species, while *Agropyron mongolicum*, *Achnatherum sibiricum*, and *Carex duriuscula* are common species in the study site.

### 4.2. Experimental Design

An outdoor pot experiment was carried out at the study site to distinguish the specific contribution of resource competition and allelopathy of *A*. *frigida* on the seedling growth of three target species. *Artemisia frigida* (AF) was utilized as the donor plant, while *L*. *chinensis* (LC), *C*. *squarrosa* (CS), and *P*. *acaulis* (PA) were selected as the recipient plants. The proportion of dry matter for each of the four species within the community is detailed in Table 2.

In our pot experiment, we utilized seedlings from four plant species, all of which were collected from the same location within the study site. Seedlings of four plant species were gathered at the stem elongation stage, as defined by the BBCH scale. To ensure homogeneous growth conditions, we selected seedlings of average size, with a coefficient of variation (CV) in plant height of less than 10% for each species. We took measures to enhance the survival rate of the seedlings by preserving the integrity of their roots during excavation. Each pot was initially watered with 500 mL, and no further irrigation was provided throughout the experiment. To maintain soil consistency across the pots, we extracted 0–30 cm topsoil from the sample plot where the plants were originally collected. This soil was then sieved, meticulously mixed, and purged of any impurities. Each pot contained six seedlings: either three from both donor and recipient plants or exclusively six from recipient plants. The pots used had a capacity of 2 gallons and dimensions of 0.24 m × 0.22 m (diameter × height). We established three biological replicates for each treatment.

**Figure 4:**
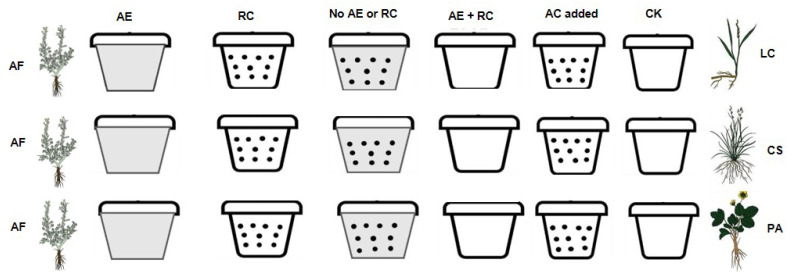
Design of outdoor pot experimental to differentiate between allelopathy and resource competition of *Artemisia frigida* in a temperate grassland in northern China. The allelopathic effect of *A*. *frigida* was eliminated by adding activated carbon to absorb allelochemicals both in the soil and air, represented by black dots on the pots. Resource competition was mitigated by introducing a PVC partition in the center of the pot, depicted as gray-shaded pots in the figure. The four plant species used in this study included LC, *Leymus chinensis*; CS, *Cleistogenes squarrosa;* AF, *Artemisia frigida;* and PA, *Potentilla acaulis*, representing the dominant species under non-grazing, light grazing, moderate grazing, and extreme grazing intensities at the study site, respectively. The experimental conditions were categorized as follows: AE (allelopathy), RC (resource competition), No AE or RC (neither allelopathy nor resource competition), AE + RC (both allelopathy and resource competition), AC added (activated carbon was added to assess its impact on plant growth), and CK (three target species planted individually).

The experimental design is illustrated in Figure 4. Our field control experiment incorporated five treatments: allelopathy of AF(AE), resource competition of AF(RC), neither allelopathy nor resource competition of AF (No AE or RC), both allelopathy and resource competition of AF (AE + RC), and the addition of activated carbon (AC added). The allelopathic effect of *A*. *frigida* was neutralized by introducing activated carbon, a method frequently employed in previous studies (represented by black-dotted pots in Figure 4) [12,13,19]. We used analytical-grade activated carbon (charcoal-activated powder extra pure, Zhejiang Minxin Ecological Technology Company, Quzhou, China), added at a concentration of 20 mL/L according to Parepa et al. [50]. The activated carbon was divided into two portions: one was mixed into the soil to absorb allelochemicals released by the roots of *A*. *frigida*, while the other was placed on the pot surface to absorb VOCs emitted by *A*. *frigida*.

Resource competition was eliminated by introducing a PVC plastic sheet into the center of the pot (gray-shaded pots in Figure 4) [34]. Three individuals from each species were planted into a pot, with each species situated on opposite sides of the plastic sheet. The sheet was affixed to the sides of each pot, ensuring that each side had equal resources and space for plant growth. We conducted a standard partition study where both roots and shoots of each species were physically separated. The impact of activated carbon on seedling growth of the three target plant species was assessed by incorporating activated carbon into the pots under monoculture conditions (white spotted pots in Figure 4). The entire experiment spanned over 2 months, from June 2019 to September 2019. All 72 pots were placed outdoors throughout the experimental period.

### 4.3. Measurements

Biomass measurements were conducted at the end of the experiment. Seedlings were divided into aboveground and belowground parts by cutting at the root collar’s apex. Roots were thoroughly washed, and both aboveground and belowground samples were all oven-dried (65 °C) for a minimum of 48 h before being weighed. We also evaluated the soil nutrient contents used for the field control experiment, and the soil description of the study site is present in Supplementary Table S1.

### 4.4. Data Analysis

The index of relative neighbor effect (*RNE*) was used to quantify the interference between *A*. *frigida* and the three target plant species, as it can overcome skewed distribution and facilitate statistical analysis compared to the commonly used index of relative competitive intensity (*RCI*) [51,52]:

*RNE* = (*P_mono_* − *P_mix_*)/*P_max_* (1)

where *P_mono_* and *P_mix_* represent the performance of four plant species under monoculture and mixed culture conditions, respectively. The maximum value between *P_mono_* and *P_mix_* is denoted as *P_max_*. For this research, we utilized aboveground biomass, belowground biomass, and root/shoot ratio as performance parameters. The *RNE* value ranged from −1 to 1, where positive and negative *RNE* values signify competitive and facilitatory effects, respectively.

Meanwhile, the index of inhibitory rate (*IR*) was used to distinguish the specific impacts of resource competition and allelopathy from *A*. *frigida* on the three target plant species:

*IR* = (1 − *T*/*C*) × 100% (2)

where *T* represents the performance of four plant species under polyculture conditions, while *C* represents the performance of four plant species under monoculture conditions. The positive and negative values of *IR* indicate inhibitory and facilitatory effects, respectively.

We calculated the indices of *IR* separately for resource competition and allelopathy, noting that the values of *T* and *C* differed between resource competition and allelopathy. To calculate the indices of *IR* under resource competition for *A*. *frigida*, *T* represents the performance of the recipient species in polyculture conditions, influenced solely by interspecific competition, with allelopathy eliminated by the addition of activated carbon (RC). Conversely, *C* represents the performance of the recipient species in monoculture conditions with the inclusion of activated carbon (AC added), ensuring that the effect of activated carbon on plant growth was counterbalanced [40].

As for calculating the indices of *IR* under allelopathy of *A*. *frigida*, *T* represents the performance of the recipient species in polyculture conditions influenced solely by allelopathy. To eliminate resource competition, a PVC partition was introduced in the center of each pot (AE). Conversely, *C* denotes the performance of recipient species in monoculture conditions (CK).

### 4.5. Statistical Analyses

This study tried to ascertain the significance of *RNE* and *IR* among target plant species and the significance of plant growth of target plant species among five treatments (AE, RC, AE + RC, No AE or RC, AC added). ANOVA is used for significance, and the LSD is used for grouping when data show significant differences among treatments. All experimental data are reported as the mean ± standard error (SE). A significance level of α = 0.05 was adopted for all analytical determinations. Statistical analyses were performed using R statistical software version 3.6.1 (R Project for Statistical Computing, Auckland, NZ). All the graphs were generated with ggplot2.

## 5. Conclusions

Overall, our findings indicate that the type and intensity of plant interference significantly vary among different species. The growth of *L*. *chinensis*, *C*. *squarrosa*, and *P*. *acaulis* is primarily affected by resource competition, allelopathy, and the combined effect of allelopathy and resource competition from *A*. *frigida*, respectively. The inhibitory effect of *A*. *frigida* on *L*. *chinensis* and *C*. *squarrosa*, coupled with its promotional effect on *P*. *acaulis*, could partially elucidate the community succession observed at the study site in relation to grazing gradients. Both inhibitory and facilitation models make a substantial contribution to the process of community succession. Our results may aid in distinguishing the mechanisms involved in plant interference and community succession within grassland under long-term grazing. Future work should focus on mutual interference among *L*. *chinensis*, *C*. *squarrosa*, and *P*. *acaulis* in plant interactions.

## Figures and Tables

**Table 1:** Interference between *Artemisia frigida* and three target plant species at the study site.

Indexes of Plant Interference	*RNE*		
	Aboveground Biomass	Belowground Biomass	Root/Shoot Ratio
AF-LC	0.28 ± 0.10 a	0.15 ± 0.04 b	−0.16 ± 0.08 b
AF-CS	0.45 ± 0.10 a	0.64 ± 0.07 a	0.30 ± 0.08 a
AF-PA	−0.08 ± 0.04 b	−0.67 ± 0.01 c	−0.64 ± 0.05 c
LC-AF	−0.27 ± 0.07 b	−0.40 ± 0.07 a	−0.18 ± 0.05 b
CS-AF	−0.64 ± 0.02 c	−0.52 ± 0.04 b	0.23 ± 0.09 a
PA-AF	0.30 ± 0.06 a	−0.59 ± 0.04 b	−0.71 ± 0.02 c

Note: Different lowercase letters in the same column indicate significant differences in the *RNE* among three target plant species. All the data are shown as mean ± SE (n = 3). AF-LC: interference of *Artemisia frigida* with *Leymus chinensis*; LC-AF: interference of *L*. *chinensis* with *A. frigida*; AF-CS: interference of *A*. *frigida* with *Cleistogenes squarrosa*; CS-AF: interference of *C*. *squarrosa* with *A*. *frigida*; AF-PA: interference of *A*. *frigida* with *Potentilla acaulis*; and PA-AF: interference of *P*. *acaulis* with *A*. *frigida*.

**Table 2:** The dry matter proportion of four plant species at the study site along with grazing gradients.

Plant Species	Dry Matter Proportion (%)			
	No Grazing	Light Grazing	Medium Grazing	Extreme Grazing
*Leymus chinensis*	35.34 ± 1.45 a	26.32 ± 1.89 b	15.53 ± 1.23 c	6.12 ± 0.78 d
*Cleistogenes squarrosa*	5.61 ± 0.32 d	43.64 ± 3.27 a	25.25 ± 2.32 b	13.42 ± 1.62 c
*Artemisia frigida*	3.26 ± 0.25 c	10.25 ± 1.67 b	38.21 ± 2.87 a	28.02 ± 2.98 a
*Potentilla acaulis*	0.08 ± 0.03 c	2.35 ± 0.08 bc	7.30 ± 1.12 b	32.75 ± 4.25 a

Note: Different lowercase letters in the same line indicate significant differences in the dry matter proportion of four plan species among four grazing intensities. All the data are shown as mean ± SE (n = 3).

## Data Availability

Data is contained within the article.

## Supplementary Materials

The following supporting information can be downloaded at: https://www.mdpi.com/article/10.3390/plants13233286/s1, Table S1: Soil description of the study site used for pot experiment.
