# Supplementary material for: The Specific Impacts of Allelopathy and Resource Competition from Artemisia frigida on the Growth of Three Plant Species in Northern China
Source: Plants (Basel). 2024 Nov 22;13(23):3286. doi: 10.3390/plants13233286 (PMC11644693; doi:10.3390/plants13233286)
Supplement: Supplementary file 1 [file plants-13-03286-s001.zip › plants-3263646-supplementary.pdf]

Table S1 Soil description of the study site used for pot experiment (mean ± SE; n=4).

Soil water content (%)	Soil porosity (%)	Soil bulk density(g/cm^3)	Soil organic carbon(g/kg)	Soil total nitrogen (g/kg)	Soil total phosphorus (g/kg)
27.66±1.23	46.12±2.6	1.42±0.06	13.23±0.89	1.48±0.04	0.45±0.02
